# BCL-X_L_ PROTAC degrader DT2216 synergizes with sotorasib in preclinical models of KRAS^G12C^-mutated cancers

**DOI:** 10.1186/s13045-022-01241-3

**Published:** 2022-03-09

**Authors:** Sajid Khan, Janet Wiegand, Peiyi Zhang, Wanyi Hu, Dinesh Thummuri, Vivekananda Budamagunta, Nan Hua, Lingtao Jin, Carmen J. Allegra, Scott E. Kopetz, Maria Zajac-Kaye, Frederic J. Kaye, Guangrong Zheng, Daohong Zhou

**Affiliations:** 1 Department of Pharmacodynamics, College of Pharmacy, University of Florida, Gainesville, FL 32610 USA; 2 Department of Medicinal Chemistry, College of Pharmacy, University of Florida, Gainesville, FL USA; 3 Department of Neuroscience, College of Medicine, University of Florida, Gainesville, FL USA; 4 Genetics and Genomics Graduate Program, Genetics Institute, College of Medicine, University of Florida, Gainesville, FL USA; 5 Department of Anatomy and Cell Biology, College of Medicine, University of Florida, Gainesville, FL USA; 6 Division of Hematology and Oncology, Department of Medicine, College of Medicine, University of Florida, Gainesville, FL USA; 7 Department of Gastrointestinal Medical Oncology, Division of Cancer Medicine, The University of Texas MD Anderson Cancer Center, Houston, TX USA

**Keywords:** KRAS^G12C^, BCL-X_L_, PROTAC, Sotorasib, Drug resistance

## Abstract

*KRAS* mutations are the most common oncogenic drivers. Sotorasib (AMG510), a covalent inhibitor of KRAS^G12C^, was recently approved for the treatment of KRAS^G12C^-mutated non-small cell lung cancer (NSCLC). However, the efficacy of sotorasib and other KRAS^G12C^ inhibitors is limited by intrinsic resistance in colorectal cancer (CRC) and by the rapid emergence of acquired resistance in all treated tumors. Therefore, there is an urgent need to develop novel combination therapies to overcome sotorasib resistance and to maximize its efficacy. We assessed the effect of sotorasib alone or in combination with DT2216 (a clinical-stage BCL-X_L_ proteolysis targeting chimera [PROTAC]) on KRAS^G12C^-mutated NSCLC, CRC and pancreatic cancer (PC) cell lines using MTS cell viability, colony formation and Annexin-V/PI apoptosis assays. Furthermore, the therapeutic efficacy of sotorasib alone and in combination with DT2216 was evaluated in vivo using different tumor xenograft models. We observed heterogeneous responses to sotorasib alone, whereas its combination with DT2216 strongly inhibited viability of KRAS^G12C^ tumor cell lines that partially responded to sotorasib treatment. Mechanistically, sotorasib treatment led to stabilization of BIM and co-treatment with DT2216 inhibited sotorasib-induced BCL-X_L_/BIM interaction leading to enhanced apoptosis in KRAS^G12C^ tumor cell lines. Furthermore, DT2216 co-treatment significantly improved the antitumor efficacy of sotorasib in vivo. Collectively, our findings suggest that due to cytostatic activity, the efficacy of sotorasib is limited, and therefore, its combination with a pro-apoptotic agent, i.e., DT2216, shows synergistic responses and can potentially overcome resistance.

**Supplementary Information:**

The online version contains supplementary material available at 10.1186/s13045-022-01241-3.

**To the Editor**,

*KRAS* mutations are the most common drivers in non-small cell lung cancer (NSCLC), colorectal cancer (CRC) and pancreatic cancer (PC) [1]. While KRAS^G12C^ inhibitors including sotorasib have shown tumor responses in a subset of NSCLC, there was reduced activity in CRC patients. To enhance its efficacy, sotorasib has been evaluated in preclinical studies using different combinations [2–4]. These combinations, however, are mostly cytostatic, limiting the potential for clinical benefit.

BCL-X_L_ is an anti-apoptotic protein that belongs to the BCL-2 family and is an important therapeutic target in multiple cancers. However, targeting BCL-X_L_ with conventional inhibitors causes severe thrombocytopenia, limiting their clinical use [5–7]. Recently, our group has reported platelet-sparing targeting of BCL-X_L_ by proteolysis targeting chimeras (PROTACs) exemplified by DT2216. DT2216 has shown promising antitumor activities in BCL-X_L_-dependent hematologic cancers as a single agent therapy and in multiple solid tumors when combined with conventional chemotherapy [8–10]. Here, we hypothesize that combining sotorasib with DT2216 could be safer and synergistic, because BCL-X_L_ is overexpressed in KRAS-mutated tumors [11].

We found that 1 µM of DT2216 can completely deplete BCL-X_L_ in different KRAS^G12C^-mutated cancer cell lines (Additional file 1: Fig. 1a-d); therefore, we used this concentration for in vitro experiments. In our study, sotorasib showed heterogeneous effects against KRAS^G12C^-mutated cancer cell lines (Fig. 1a; Additional file 1: Fig. 2a). Moreover, sotorasib caused only partial reduction in viability in the sensitive cell lines (referred to as partially sensitive cell lines). Interestingly, a combination of sotorasib and DT2216 synergistically reduced viability of partially sensitive cell lines, as well as enhanced inhibition of colony formation and apoptosis induction compared to sotorasib monotherapy (Fig. 1a–d). We further evaluated whether or not the inhibition of other BCL-2 anti-apoptotic proteins could enhance the efficacy of sotorasib. We found that the inhibition of BCL-X_L_, but not BCL-2 or MCL-1, can sensitize all the three partially sensitive cell lines to sotorasib treatment (Additional file 1: Fig. 2b). Moreover, non-KRAS^G12C^-mutated cell lines did not respond to sotorasib treatment alone as well as in combination with DT2216 (Additional file 1: Fig. 2c). Of note, the synergistic effect of sotorasib + DT2216 combination was significantly abrogated in the presence of a pan-caspase inhibitor (QVD-OPh), indicating caspase-mediated apoptosis (Additional file 1: Fig. 3). In addition, DT2216 could not synergize with a MEK inhibitor (selumetinib) in the KRAS^G12C^-mutated cancer cell lines that did not respond to sotorasib + DT2216 combination (Additional file 1: Fig. 4). These results suggest that DT2216 can potently enhance the efficacy of sotorasib in KRAS^G12C^-mutated cancer cells which are partially sensitive to sotorasib monotherapy.

**Fig. 1:**
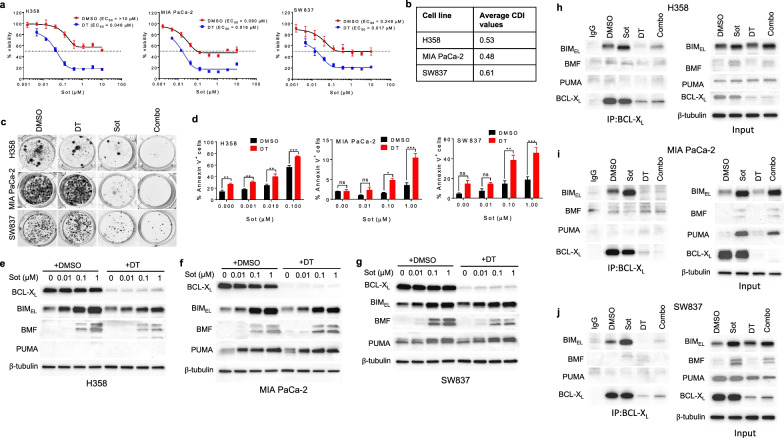
A combination of sotorasib and DT2216 has synergistic antitumor activity through suppression of BCL-X_L_/BIM interaction in KRAS^G12C^-mutated cancer cell lines. **a** Viability of KRAS^G12C^-mutated H358 NSCLC, MIA PaCa-2 PC and SW837 CRC cell lines after they were treated with increasing concentrations of sotorasib (Sot) in threefold increments with either DMSO or DT2216 (DT, 1 µM) for 72 h. The data are presented as percentage viability relative to control (mean ± SD; *n* = 6 replicate cell cultures) as measured by MTS assay. **b** CDI values were calculated at different concentrations of Sot used in combination with 1 µM of DT, and their averages are shown in the table for H358, MIA PaCa-2 and SW837 cell lines. CDI < 0.7 indicates significant synergistic effect. CDI < 1 indicates synergistic effect, CDI = 1 indicates additive effect, and CDI > 1 indicates antagonistic effects. EC_50_, half-maximal effective concentration (equivalent to IC_50_ or half-maximal inhibitory concentration); CDI, coefficient of drug interaction. **c** colony formation in indicated cell lines after they were treated with DT (1 µM), Sot (0.1 µM), or a combination of the two (Combo) for 10–14 days followed by crystal violet staining. **d** Apoptosis in the cell lines after they were treated with indicated concentrations of Sot with either DMSO or DT (1 µM) for 48 h (H358) or 72 h (MIA PaCa-2 and SW837). The data are presented as percentage Annexin V^+^ (apoptotic) cells in total cell population (mean ± SEM) as measured by Annexin V/PI staining using flow cytometry. Statistical significance was determined by one-way ANOVA and Tukey’s multiple comparison test. *p < 0.05, **p < 0.01, ***p < 0.001. ns, not significant. **e**–**g** Immunoblot analysis of BCL-X_L_, BIM, BMF and PUMA in H358 (**e**), MIA PaCa-2 (**f**) and SW837 (**g**) cell lines after they were treated with indicated concentrations of Sot with either DMSO or DT (1 µM) for 24 h. Immunoblots detect three isoforms of BIM, i.e., short isoform (BIM_S_), long isoform (BIM_L_) and extra-long isoform (BIM_EL_). Among them, BIM_EL_ is the major isoform and is shown here. Densitometry graphs of selected immunoblots normalized to equal loading control β-tubulin are shown in Additional file 1: Fig. 8a-c. **h**–**j** Immunoprecipitation analysis of BCL-X_L_ in H358 (**h**), MIA PaCa-2 (**i**) and SW837 (**j**) cell lines after they were treated with Sot (0.1 µM), DT (1 µM) or Combo for 24 h, and the immunoprecipitated as well as input samples were subjected to immunoblot analysis of BIM, BMF, PUMA and BCL-X_L_. β-tubulin was used as an equal loading control

**Fig. 2:**
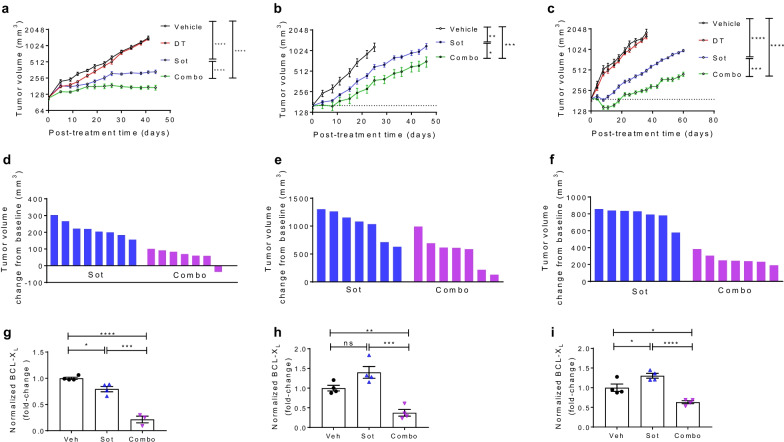
BCL-X_L_ degradation with DT2216 enhances the antitumor efficacy of sotorasib in KRAS^G12C^-mutated xenograft models. **a**–**c** Tumor volume changes in H358 (**a**), MIA PaCa-2 (**b**) and SW837 (**c**) xenografts after they were treated with vehicle, DT2216 (DT, 15 mg/kg, every four days [q4d], i.p.), sotorasib (Sot, 10 mg/kg, 5 days a week, p.o.) or a combination of the two (Combo). Data are presented as mean ± SEM (*n* = 7–8 mice at the start of treatment). Statistical significances in **a**–**c** were determined by two-sided Student’s *t*-test at day-41, day-25 and day-25 in H358, MIA PaCa-2 and SW837 xenografts, respectively. *p < 0.05, **p < 0.01, ***p < 0.001, ****p < 0.0001. **d**–**f** Changes in tumor volumes of individual mice in H358 (**d**), MIA PaCa-2 (**e**) and SW837 (**f**) xenografts at the end of treatment calculated as: tumor volume at the end of treatment − baseline tumor volume. **g**–**i** Densitometric analysis showing BCL-X_L_ protein levels as determined by immunoblotting in H358 (**g**), MIA PaCa-2 (**h**) and SW837 (**i**) xenograft tumors at the end of treatments as in **a**–**c** (*n* = 4 mice per group). The representative immunoblots are shown in Additional file 1: Fig. 12a

Next, we elucidated the mechanism involved in the DT2216 + sotorasib synergistic activity. DT2216 co-treatment with sotorasib was not able to enhance or prolong the inhibition of KRAS signaling (Additional file 1: Fig. 5a-h; Additional file 1: Fig. 6). Therefore, we hypothesized that sotorasib might induce apoptotic priming through the stabilization of BH3-only pro-apoptotic proteins (e.g., BIM, BMF and PUMA). We observed a concentration-dependent upregulation of BIM and BMF after sotorasib treatment in all the partially sensitive cell lines, while PUMA was also upregulated in MIA PaCa-2 and SW837 (Fig. 1e–g; Additional file 1: Fig. 7a-d and 8a-d). Sotorasib or sotorasib + DT2216 had no considerable effect on other BCL-2 proteins (Additional file 1: Fig. 7a-d). Further, sotorasib + DT2216 combination led to a pronounced increase in cleaved caspase-3 and cleaved PARP levels in partially sensitive cell lines indicating apoptosis induction (Additional file 1: Fig. 7a-c). We observed a decrease in p-BIM (S69) levels after sotorasib treatment (Additional file 1: Fig. 9a, b), which might be attributed to BIM stabilization as ERK activation is known to induce BIM (S69) phosphorylation and degradation (12). In addition, sotorasib led to a concentration-dependent upregulation of *BCL2L11* (BIM coding gene) (Additional file 1: Fig. 9c). Next, we found that sotorasib selectively induces BCL-X_L_ interaction with BIM, which was disrupted upon BCL-X_L_ degradation with DT2216 (Fig. 1h-j). These results suggest that sotorasib induces apoptotic priming that can be exploited by DT2216 to induce apoptosis in KRAS^G12C^-mutated cancer cells.

Finally, we investigated the efficacy of sotorasib + DT2216 combination in mouse xenografts. As expected, DT2216 alone had no significant effect on tumor growth. The DT2216 + sotorasib combination led to significant tumor inhibition compared to sotorasib monotherapy (Fig. 2a–f). The combination treatment was quite safe as indicated by no significant change in mouse body weights, as well as no clinically significant decrease in blood cell counts was seen (Additional file 1: Fig. 10a-c; Additional file 1: Fig. 11a, b). We also confirmed BCL-X_L_ degradation and KRAS engagement with DT2216 and sotorasib treatments, respectively (Fig. 2g–i; Additional file 1: Fig. 12a). In addition, sotorasib-mediated inhibition of ERK was associated with BIM accumulation in MIA PaCa-2 xenograft tumors leading to an increase in cleaved caspase-3 and cleaved PARP in combination-treated tumors (Additional file 1: Fig. 12b, c). Further, IHC staining showed a considerable decrease in Ki67 and a significant increase in cleaved caspase-3 in combination-treated H358 tumors (Additional file 1: Fig. 12d), which was consistent with tumor growth inhibition (Fig. 2a, d).

In conclusion, our studies show that DT2216 enhances the therapeutic efficacy of sotorasib which warrants clinical testing of this combination, particularly in KRAS^G12C^-mutated CRC patients who otherwise derive minimal benefit from sotorasib monotherapy.

## Supplementary Information

**Additional file 1**. Materials and Methods, Supplementary Figures, and Supplementary Table.

## Data Availability

All data generated or analyzed during this study are included in this published article or its supplementary information files. The raw datasets are available from the corresponding authors on reasonable request.

## Abbreviations

NSCLC: Non-small cell lung cancer
CRC: Colorectal cancer
PC: Pancreatic cancer
RTK: Receptor tyrosine kinase
BCL-X_L_: B-cell lymphoma extra-large
PROTAC: Proteolysis targeting chimera
IHC: Immunohistochemistry
