# Supplementary material for: BCL-XL PROTAC degrader DT2216 synergizes with sotorasib in preclinical models of KRASG12C-mutated cancers
Source: J Hematol Oncol. 2022 Mar 9;15:23. doi: 10.1186/s13045-022-01241-3 (PMC8905794; doi:10.1186/s13045-022-01241-3)
Supplement: Supplementary file 1 — Additional file 1. Materials and Methods, Supplementary Figures, and Supplementary Table. [file 13045_2022_1241_MOESM1_ESM.docx]

**ADDITIONAL FILE 1: Materials and Methods, Supplementary Figures, and Supplementary Table**

**Khan S et al. BCL-X_L_ PROTAC degrader DT2216 synergizes with sotorasib in preclinical models of KRAS^G12C^-mutated cancers**

| **Supplementary Item** | **Title** |
| --- | --- |
| Materials and Methods | Materials and Methods |
| Supplementary Fig. 1 | DT2216 causes dose-dependent degradation of BCL-X_L_ in KRAS^G12C^-mutated cancer cell lines |
| Supplementary Fig. 2 | Sotorasib+DT2216 combination is selective against KRAS^G12C^-mutated tumor cell lines |
| Supplementary Fig. 3 | A pan-caspase inhibitor significantly abrogates the apoptosis induction by sotorasib+DT2216 in KRAS^G12C^-mutated cancer cell lines |
| Supplementary Fig. 4 | DT2216 does not synergize with MEK inhibitor (Selumetinib) in Sotorasib-resistant cell lines |
| Supplementary Fig. 5 | DT2216 co-treatment has no further effect on KRAS downstream signaling in KRAS^G12C^-mutated cancer cell lines |
| Supplementary Fig. 6 | DT2216 does not prolong KRAS signaling inhibition when combined with sotorasib |
| Supplementary Fig. 7 | Sotorasib increases apoptotic priming through stabilization of pro-apoptotic BH3-only proteins in KRAS^G12C^-mutated cancer cell lines |
| Supplementary Fig. 8 | Densitometric analysis of BCL-X_L_ and BIM in KRAS^G12C^-mutated cancer cell lines after treatment with sotorasib and/or DT2216 |
| Supplementary Fig. 9 | Sotorasib upregulates BIM at both transcriptional and post-translational levels |
| Supplementary Fig. 10 | Sotorasib+DT2216 combination has no effect on mouse body weights |
| Supplementary Fig. 11 | Sotorasib+DT2216 combination does not cause considerable reductions in blood cell counts |
| Supplementary Fig. 12 | DT2216 effectively degrades BCL-X_L_ and sotorasib+DT2216 combination induces apoptosis *in vivo* |
| Supplementary Table 1 | Antibodies used in immunoblotting |
| Supplementary References | Supplementary References |

**MATERIALS AND METHODS**

**Cell culture**

NCI-H358 (H358, Cat. No. CRL-5807), NCI-H23 (H23, Cat. No. CRL-5800), Calu-1 (Cat. No. HTB-54), NCI-H1792 (H1792, Cat. No. CRL-5895), NCI-H2122 (H2122, Cat. No. CRL-5985), A549 (Cat. No. CCL-185), NCI-H1299 (H1299, Cat. No. CRL-5803), MIA PaCa2 (Cat. No. CRL-1420) and SW837 (Cat. No. CCL-235) human cancer cell lines were purchased from the American Type Culture Collection (ATCC, Manassas, VA). All the cell lines except MIA PaCa-2 were cultured in RPMI-1640 medium (Cat. No. 22400–089, Thermo Fisher Scientific [Thermo Fisher], Waltham, MA). MIA PaCa-2 cell line was cultured in Dulbecco’s modified Eagle’s medium (DMEM) (Cat. No. 12430-062, Thermo Fisher). All culture media were supplemented with 10% heat-inactivated fetal bovine serum (FBS, Cat. No. S11150H, Atlanta Biologicals, GA), 100 U/mL penicillin and 100 µg/mL streptomycin (Pen-Strep, Cat. No. 15140122, Thermo Fisher). All cultures were confirmed for Mycoplasma negativity using the MycoAlert Mycoplasma Detection Kit (Cat. No. LT07–318, Lonza, Basel, Switzerland). All the cell lines were maintained in a humidified incubator at 37° C and 5% CO_2_.

**Chemical compounds**

DT2216 was synthesized in Dr. Guangrong Zheng’s laboratory (University of Florida, Gainesville, FL) according to the previously described protocol (1). Sotorasib (Cat. No. HY-114277), Selumetinib (Cat. No. HY-50706) and QVD-OPh (Cat. No. HY-12305) were purchased from MedChemExpress (Monmouth Junction, NJ). A1155463 (Cat. No. S7800), A1331852 (Cat. No. S7801), ABT199 (Cat. No. S8048), S63845 (Cat. No. S8383), and ABT263 (Cat. No. S1001) were purchased from SelleckChem (Houston, TX). All the compounds were dissolved in DMSO at 10 mM stock solution for *in vitro* assays.

**MTS cell viability assay**

Cells were seeded in 96-well plates at a density of 3,000-5,000 cells per well. After overnight incubation, different concentrations of the drugs were added in 3-6 replicates to the plates. The MTS assay was performed according to the manufacturer’s protocol (Cat. No. G-111, Promega, Madison, WI) and as described previously (1, 2). The absorbance was recorded at 490 nm using Biotek’s Synergy Neo2 multimode plate reader (Biotek, Winooski, VT). EC_50_ values were determined using GraphPad Prism software (GraphPad Software, La Jolla, CA).

**Colony formation assay**

A total of 1,000-10,000 cells per well were seeded in 12-well plates. After overnight incubation, the cells were treated with sotorasib, DT2216, or a combination of the two for 10-14 days. Fresh treatment-containing medium was added to the plates every four days. At the end, the cells were fixed with absolute methanol, and then stained with 0.1% crystal violet solution. The images were captured using ChemiDoc MP Imaging System (Bio-Rad, Hercules, CA).

**Annexin-V/PI apoptosis assay**

Cells were seeded in 12-well plates at a density of 1×10^5^ cells per well and were treated with sotorasib, DT2216, or the combination of the two for 48-72 h. After incubation, the cells were collected by trypsinization and were stained with Annexin V-Alexa Fluor 647 (1:50, Cat. No. 640912, BioLegend, San Diego, CA) and propidium iodide (PI, 1 μg/mL, Cat. No. 421301, BioLegend) for 30 min at room temperature. The samples were analyzed using flow cytometer (Aurora, Cytek Biosciences, Fremont, CA) and the SpectraFlo software (Repligen, Waltham, MA). A minimum of 10,000 events were recorded for each sample. The percentage of apoptotic cells was defined as the sum of Annexin V-positive/ PI-negative and Annexin V-positive/ PI-positive cells.

**Immunoblotting**

Protein lysates were prepared from cells using RIPA lysis buffer (Cat. No. BP-115DG, Boston Bio Products, Ashland, MA) supplemented with protease and phosphatase inhibitor cocktail (Cat. No. PPC1010, Sigma-Aldrich, St. Louis, MO) as described previously (1). Briefly, an equal amount of proteins (20-40 µg/lane) were loaded to a precast gel and transferred onto PVDF membranes. The membranes were blocked with 5% (w/v) non-fat dry milk in TBS-T buffer, and subsequently probed with primary antibodies overnight at 4 °C. After washing with TBST, the membranes were incubated with horseradish peroxidase (HRP)-linked secondary antibody for 1-2 h at room temperature. Finally, the membranes were incubated with chemiluminescent HRP substrate (Cat. No. WBKLS0500, MilliporeSigma, Billerica, MA), and were recorded using the ChemiDoc MP Imaging System. The densitometric analysis of immunoblots was performed using Image J software. The primary antibody details are provided in Supplementary Table-1.

**RNA extraction and quantitative real-time PCR**

RNA was isolated from cells using RNeasy Mini Kit (Cat. No. 74106, Qiagen, Hilden, Germany). A total of 1 µg of RNA was converted into cDNA using high-capacity cDNA reverse transcription kit (Cat. No. 4368813, Applied Biosystems, Foster City, CA) as per the manufacturer’s instructions. Gene expression of *BCL2L11* was then quantified using gene specific primers (Forward: CAA GAG TTG CGG CGT ATT GGA G; Reverse: ACA CCA GGC GGA CAA TGT AAC G), and SYBR Green Master-Mix (Cat. No. 4385617, Applied Biosystems, Foster City, CA). The expression of *GAPDH* was used for normalization and the level of gene expression in DMSO control cells was used as a baseline. Fold-change in gene expression was calculated using the ΔΔCT method.

**Co-immunoprecipitation**

Cell pellets were lysed in the Pierce IP lysis buffer (Cat. No. 87787; Thermo Fisher) supplemented with protease and phosphatase inhibitors as described previously (1, 2). The supernatants were collected and precleared by incubating with 1 µg of mouse anti-IgG (Cat. No. sc-2025; Santa Cruz Biotechnology [SCB], Dallas, TX) and 20 µL of protein A/G-PLUS agarose beads (Cat. No. sc-2003; SCB) for 30 min at 4 °C. The supernatants containing 1 mg of protein were incubated with 2 µg of anti-BCL-X_L_ (Cat. No. sc-56021; SCB) or anti-IgG antibody overnight followed by incubation with 25 µL protein A/G agarose beads for 1-2 h at 4 °C. Thereafter, the immunoprecipitates were collected by centrifugation, washed three times with IP lysis buffer, mixed with 50 µL of Laemmli’s SDS-buffer, denatured and then subjected to immunoblot analysis for BIM, BMF, PUMA and BCL-X_L_. Anti-rabbit HRP-conjugated Fc fragment specific secondary antibody (Cat. No. 111-035-046, dilution 1:10000, Jackson ImmunoResearch, West Grove, PA) was used to detect immune complexes in immunoblotting.

**Animal studies**

NOD-*scid* IL2Rgamma^null^ (NSG) mice aged 5-6 weeks were purchased from the Jackson Laboratory (Stock No. 005557, Bar Harbor, ME). H358, MIA PaCa-2 or SW837 tumor cells at the density of 5×10^6^ per mouse in 50% Matrigel (Cat. No. 356237, Corning, Corning, NY) in culture medium or PBS were injected subcutaneously (s.c.) into the right flank region of the mice as described previously (1, 2). Tumor size was measured twice a week with digital calipers and tumor volume was calculated using the formula (Length×Width^2^×0.5). The mice were randomized into different treatment groups when the tumors reached 100-200 mm^3^. Mice were treated with vehicle, sotorasib (10 mg/kg, 5 days a week, p.o.), DT2216 (15 mg/kg, every four days [q4d], i.p.) and a combination of sotorasib and DT2216. Sotorasib was formulated in 10% (v/v) DMSO, and 90% of 20% (w/v) Captisol (Cat. No. NC0604701, Cydex Pharmaceuticals a Ligand Company, San Diego, CA) in normal saline and DT2216 was formulated in 50% phosal 50 PG, 45% miglyol 810N and 5% polysorbate 80. Mice were euthanized when they became moribund, or their tumor sizes reached a humane endpoint as per Institutional Animal Care and Use Committee (IACUC) policy. For euthanasia, animals were sacrificed by CO_2_ suffocation followed by cervical dislocation. The tumors were subsequently harvested, lysed and used for immunoblot analysis. All the animal procedures were performed in accordance with the rules of IACUC at the University of Florida.

**Complete blood-cell counts (CBCs)**

Approximately 50 µl of blood was collected from each mouse in EDTA-treated tubes *via* the *submandibular plexus*. The blood was immediately used for CBCs using an automated hematology analyzer HEMAVET 950FS (Drew Scientific Inc., Miami Lakes, FL). The data were expressed as number of different blood cells or platelets per µL of blood.

**Pharmacodynamic (PD) analysis**

MIA PaCa-2 xenografts were established in NSG mice as described above, and they were randomized into different treatment groups when the tumors reached ~400 mm^3^. Mice were treated with vehicle, sotorasib, and a combination of sotorasib and DT2216 at the abovementioned dosages. The mice were euthanized 24 h after second dose of DT2216 and/or 6 h after the sixth dose of sotorasib. The tumors were harvested from each mouse and protein lysates were prepared, and then subjected to immunoblotting analysis of BCL-X_L_, RAS, p-ERK, ERK, p-AKT, AKT, p-S6, S6, BIM, fCaspase-3, cCaspase-3, fPARP and cPARP.

**Immunohistochemistry**

Mouse tumor tissues were fixed in 4% paraformaldehyde for 24 h, and then transferred to 70% ethanol next day. Paraffin embedding, sectioning and staining were performed by the Molecular Pathology Core (University of Florida). After antigen retrieval and blocking, tissue sections were incubated with rabbit anti-human cleaved caspase-3 (Cat. No. 9664, dilution 1:600, Cell Signaling Technology, Danvers, MA) or mouse anti-human Ki67 (Cat. No. M7240, dilution 1:170, Agilent, Santa Clara, CA) antibody for 1 h followed by incubation with HRP-conjugated secondary antibody for 30 min. The images were taken at 200x magnification using EVOS XL Core microscope (Thermo Fisher).

**Statistical Analysis**

For analysis of the means of three or more groups, analysis of variance (ANOVA) tests were performed. In the event that ANOVA justified post-hoc comparisons between group means, the comparisons were conducted using Tukey’s multiple-comparisons test. A two-sided unpaired Student’s *t*-test was used for comparisons between the means of two groups. *P* <0.05 was considered to be statistically significant. The coefficient of drug interaction (CDI) was calculated as: CDI = AB/ (A × B), where AB is the ratio of percentage viable cells in the combination groups to control group, while A and B are the ratio of the percentage viable cells in single agent group to control group. CDI <1 indicates synergistic effect, CDI = 1 indicates additive effect and CDI > 1 indicates antagonistic effects. CDI <0.7 indicates significant synergistic effect.

**
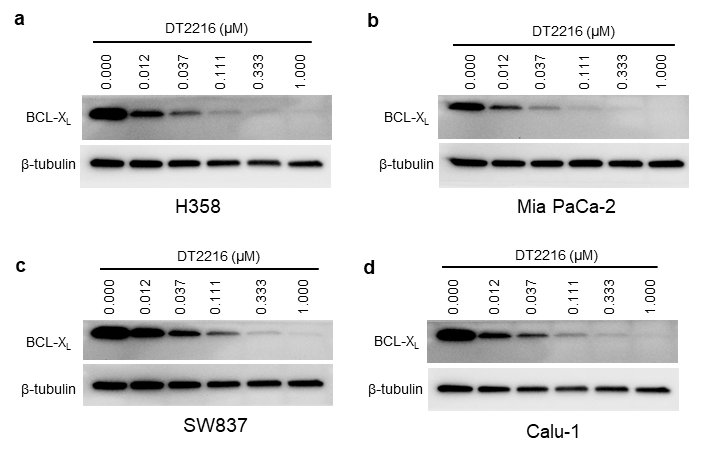
**

**Supplementary Fig. 1. DT2216 causes dose-dependent degradation of BCL-X_L_ in KRAS^G12C^-mutated cancer cell lines.** Immunoblot analysis of BCL-X_L_ in H358 (a), MIA PaCa-2 (b), SW837 (c) and Calu-1 (d) cell lines after they were treated with indicated concentrations of DT2216 for 24 h.

**
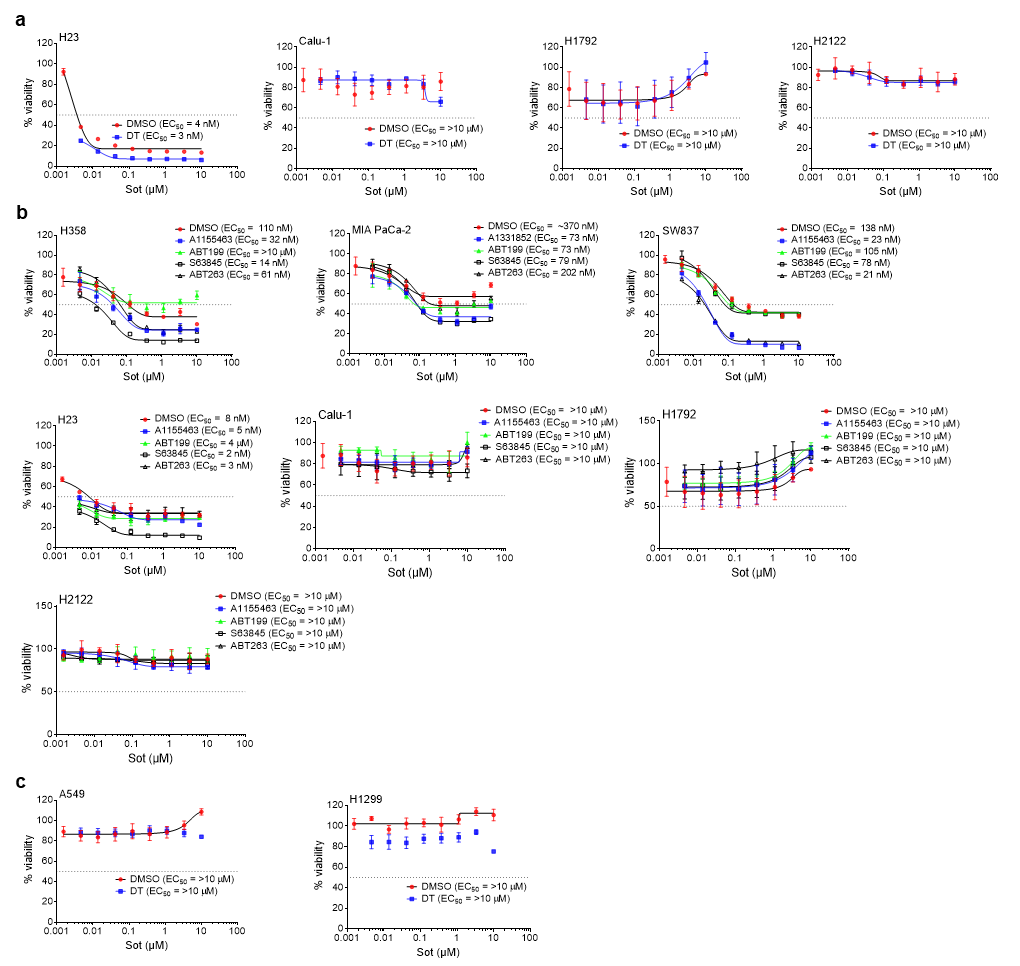
Supplementary Fig. 2. Sotorasib+DT2216 combination is selective against KRAS^G12C^-mutated tumor cell lines. a,** Viability of different KRAS^G12C^-mutated NSCLC cell lines after they were treated with increasing concentrations of sotorasib (Sot) in threefold increments with either DMSO or DT2216 (DT, 1 µM) for 72 h, except H23 cells which were treated for 6 days. **b,** Viability of different KRAS^G12C^-mutated cell lines after they were treated with increasing concentrations of Sot in threefold increments with either DMSO, BCL-X_L_ selective inhibitors A1155463/A1331852 (1 µM), BCL-2 selective inhibitor ABT199 (1 µM), MCL-1 selective inhibitor S63845 (1 µM) or BCL-X_L_/2 dual inhibitor ABT263 (1 µM) for 72 h. **c,** Viability of non-KRAS^G12C^-mutated A549 and H1299 NSCLC cell lines after they were treated with increasing concentrations of Sot in threefold increments with either DMSO or DT (1 µM) for 72 h. The data are presented as percentage viability relative to control (mean ± SD; *n* = 6 and 3 replicate cultures in **a** and **b-c**, respectively).


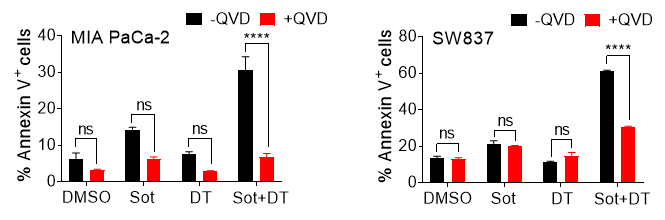


**Supplementary Fig. 3. A pan-caspase inhibitor significantly abrogates the apoptosis induction by sotorasib+DT2216 in KRAS^G12C^-mutated cancer cell lines.** Apoptosis in MIA PaCa-2 (left panel) and SW837 (right panel) cell lines was analyzed after the cells were pre-treated with 10 µM of QVD-OPh (QVD, a pan-caspase inhibitor) for 3 h followed by treatment with sotorasib (Sot, 1 µM) or DT2216 (DT, 1 µM) or a combination of Sot+DT for 72 h. The data are presented as percentage Annexin V^+^ (apoptotic) cells in total cell population (mean ± SEM) as measured by Annexin V/PI staining using flow cytometry. Statistical significance was determined by one-way ANOVA and Tukey’s multiple comparison test. **** *p* <0.001; ns, not significant.


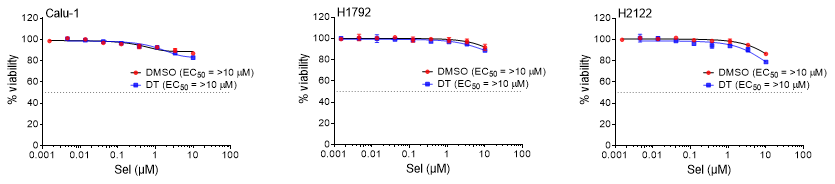


**Supplementary Fig. 4. DT2216 does not synergize with MEK inhibitor (Selumetinib) in Sotorasib-resistant cell lines.** Viability of Calu-1, H1792 and H2122 NSCLC cell lines was determined after they were treated with increasing concentrations of selumetinib (Sel) in threefold increments with either DMSO or DT2216 (DT, 1 µM) for 72 h.

**
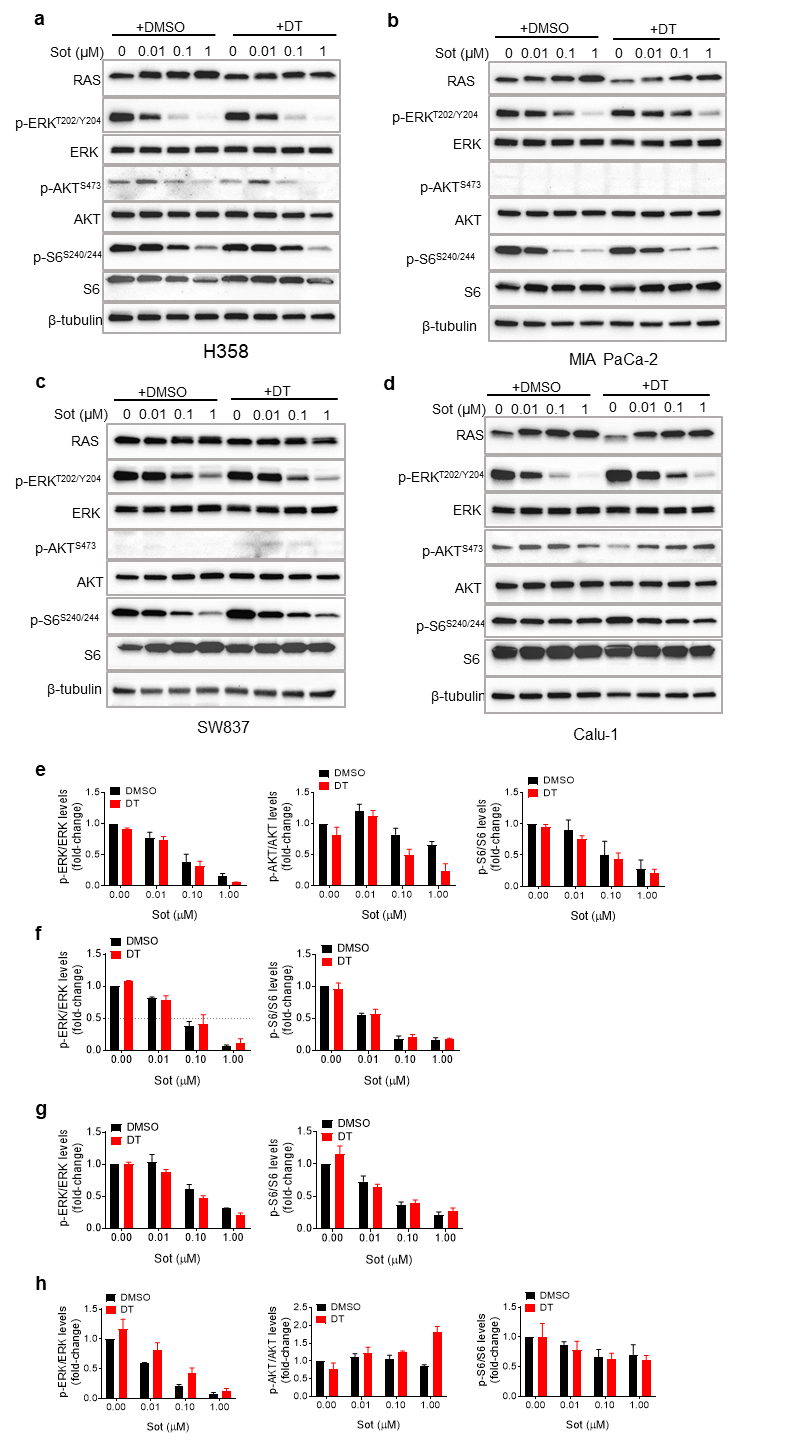
**

**Supplementary Fig. 5. DT2216 co-treatment has no further effect on KRAS downstream signaling in KRAS^G12C^-mutated cancer cell lines. a-d,** Immunoblot analysis of RAS, phosphorylated (p)-ERK (p-ERK), ERK, p-AKT, AKT, p-S6 and S6 in H358 (a), MIA PaCa-2 (b), SW837 (c) and Calu-1 (d) cell lines after they were treated with indicated concentrations of sotorasib (Sot) with either DMSO or DT2216 (DT, 1 µM) for 24 h. **e-h**, Densitometric analysis of selected immunoblots for H358 (e), MIA PaCa-2 (f), SW837 (g) and Calu-1 (h) normalized to equal loading control β-tubulin in **a-d (**mean ± SEM, *n* = 3 independent experiments).

**
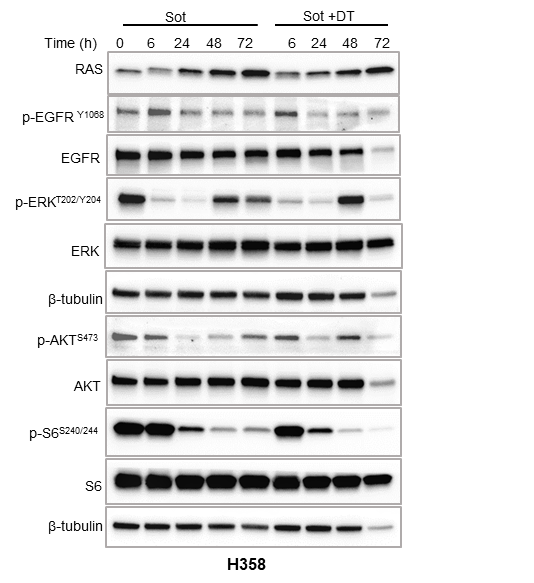
**

**Supplementary Fig. 6. DT2216 does not prolong KRAS signaling inhibition when combined with sotorasib.** Immunoblot analysis of RAS, p-EGFR, EGFR, p-ERK, ERK, p-AKT, AKT, p-S6 and S6 in H358 NSCLC cells after they were treated with sotorasib (Sot, 0.1 µM) alone or with DT2216 (DT, 1 µM) for 0, 6, 24, 48 and 72 h.

**
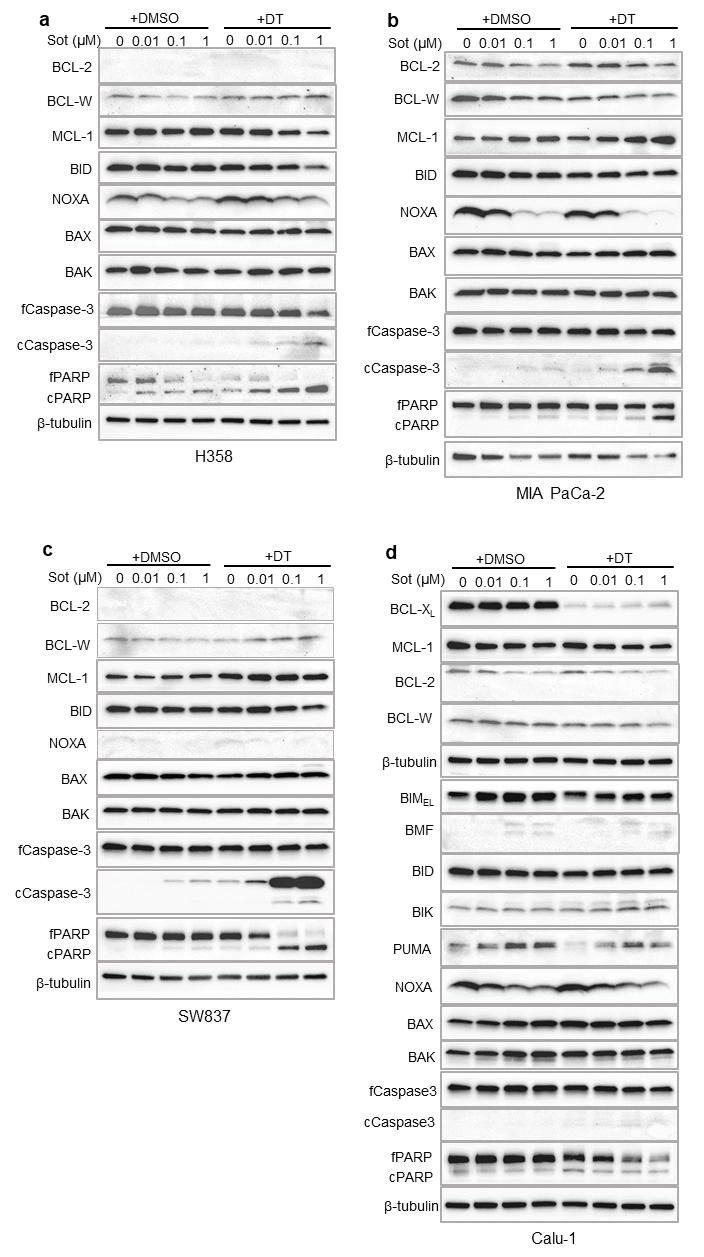
**

**Supplementary Fig. 7. Sotorasib increases apoptotic priming through stabilization of pro-apoptotic BH3-only proteins in KRAS^G12C^-mutated cancer cell lines. a-d,** Immunoblot analysis of indicated proteins in H358 (a), MIA PaCa-2 (b), SW837 (c) and Calu-1 (d) cell lines after they were treated with indicated concentrations of sotorasib (Sot) with either DMSO or DT2216 (DT, 1 µM) for 24 h.

**
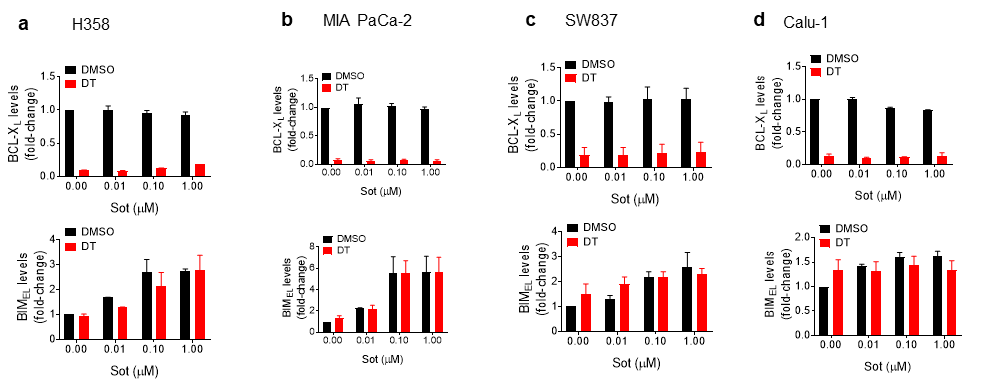
Supplementary Fig. 8. Densitometric analysis of BCL-X_L_ and BIM in KRAS^G12C^-mutated cancer cell lines after treatment with sotorasib and/or DT2216.** Densitometric analysis of selected immunoblots in H358 (a), MIA PaCa-2 (b), SW837 (c) and Calu-1 (d) cell lines normalized to equal loading control β-tubulin in **Fig. 1e-g** and **Supplementary Fig. 7d (**mean ± SEM, *n* = 2 independent experiments**).**

**
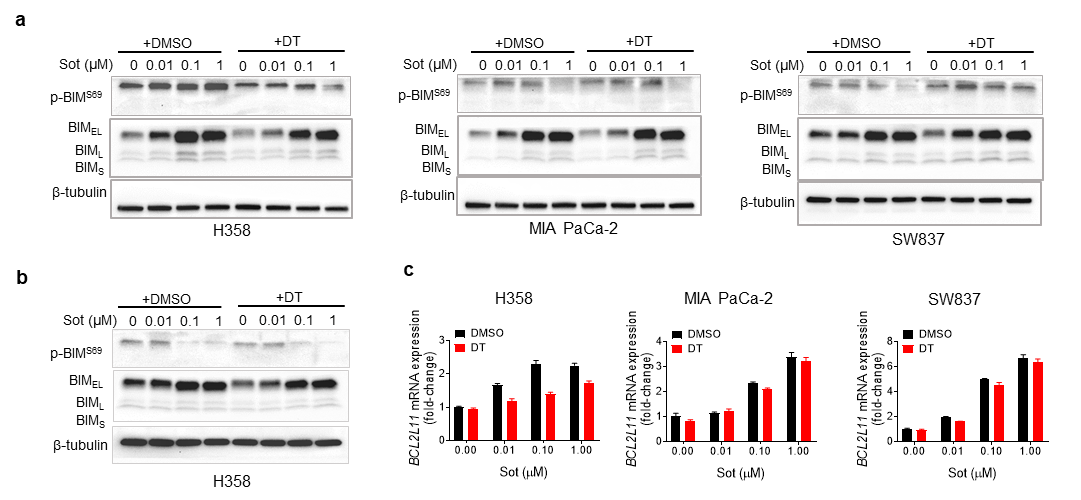
Supplementary Fig. 9. Sotorasib upregulates BIM at both transcriptional and post-translational levels. a,** Immunoblot analysis of p-BIM S69 and BIM in H358, MIA PaCa-2 and SW837 cells after they were treated with indicated concentrations of sotorasib (Sot) with either DMSO or DT2216 (DT, 1 µM) for 24 h. **b,** Immunoblot analysis of p-BIM S69 and BIM in H358 cells after they were treated with indicated concentrations of Sot with either DMSO or DT (1 µM) for 6 h. **c,** qPCR analysis of *BCL2L11* (BIM coding gene) in H358, MIA PaCa-2 and SW837 cells after they were treated with indicated concentrations of Sot alone or with DT (1 µM) for 24 h.

**
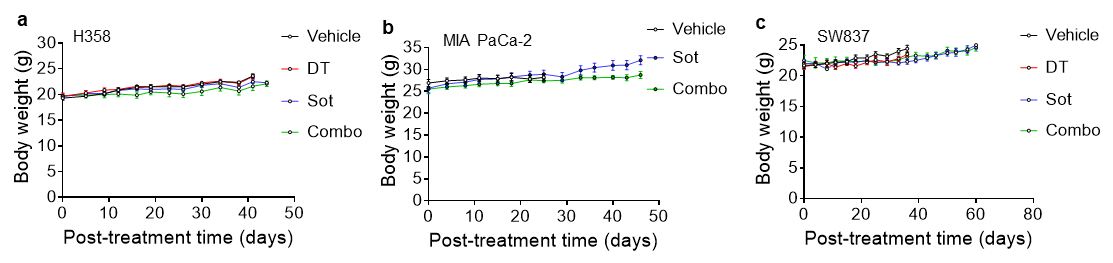
Supplementary Fig. 10. Sotorasib+DT2216 combination has no effect on mouse body weights.** Body weight changes in H358 (a), MIA PaCa-2 (b) and SW837 (c) xenografted mice after they were treated with Vehicle, DT2216 (DT, 15 mg/kg, q4d, i.p.), sotorasib (Sot, 10 mg/kg, 5 days a week, p.o.) or a combination of the two (Combo) as in **Fig.** **2a-c**. Data are presented as mean ± SEM (n = 7-8 mice at the start of treatment).

**
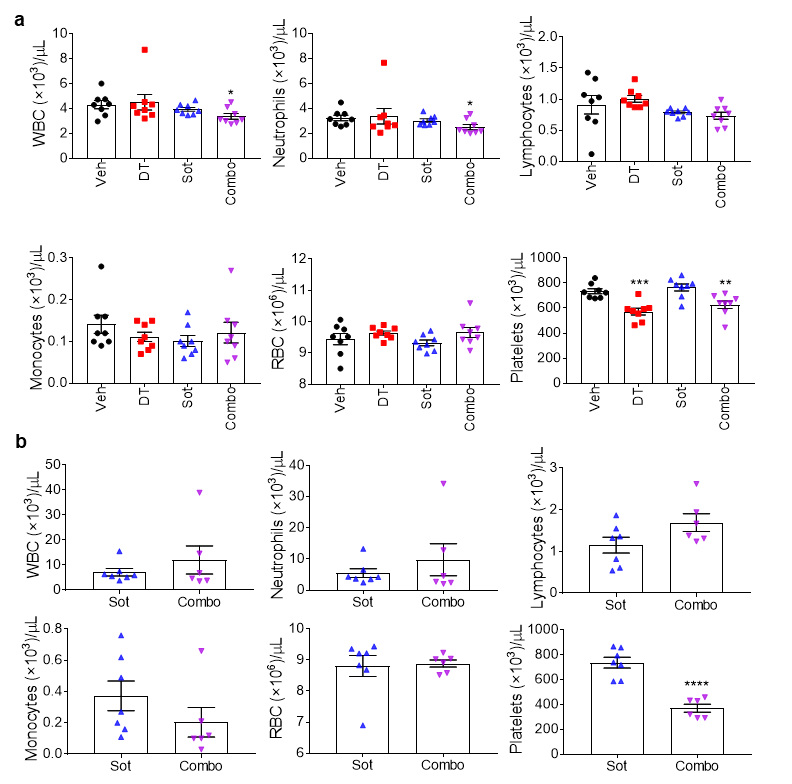
Supplementary Fig. 11. Sotorasib+DT2216 combination does not cause considerable reductions in blood cell counts. a, b,** Different blood cell counts in H358 xenografted mice 24 h after first (a) and last treatments (b) as in **Fig. 2a**.

**
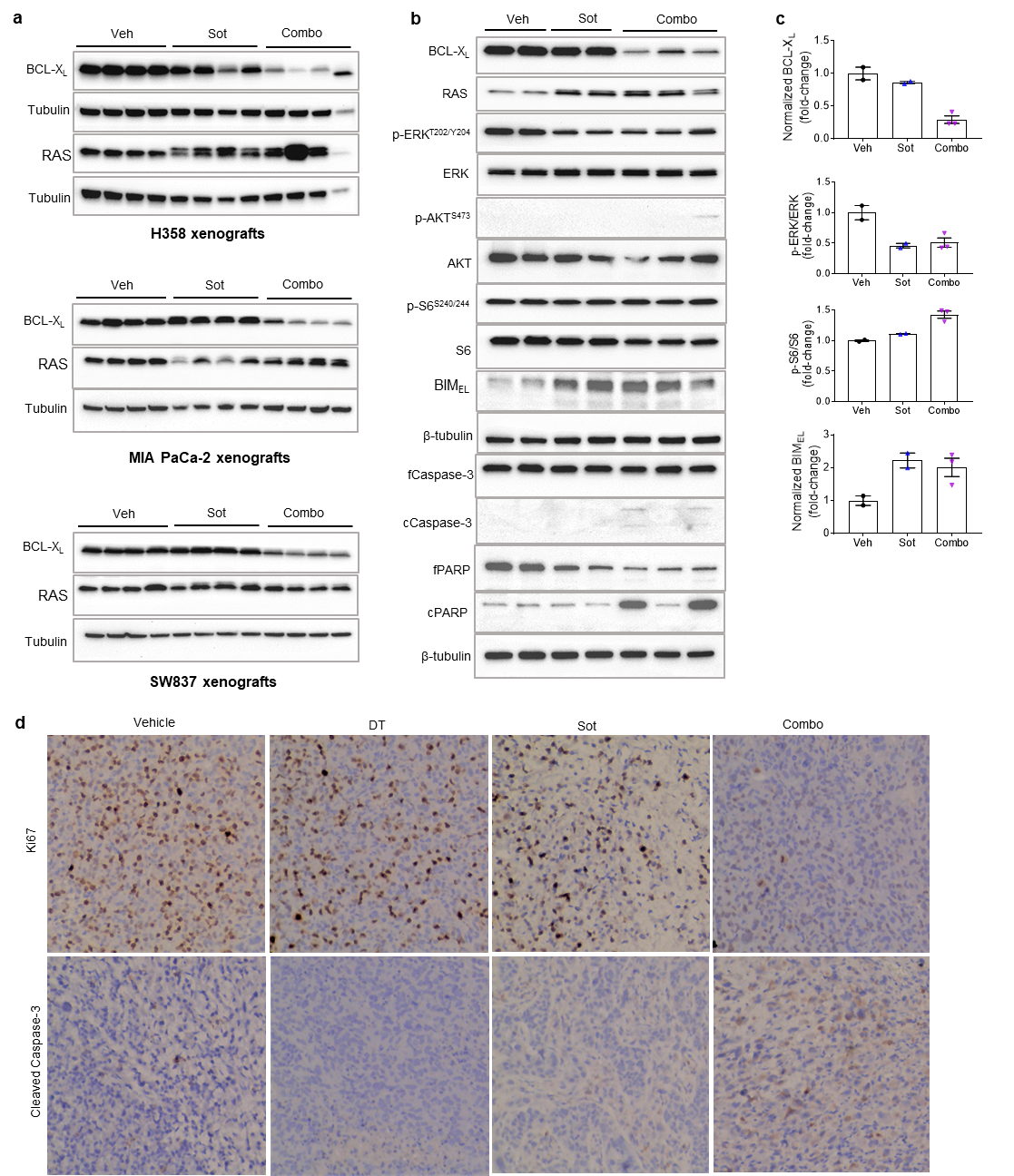
 Supplementary Fig. 12. DT2216 effectively degrades BCL-X_L_ and sotorasib+DT2216 combination induces apoptosis *in vivo*. a,** Immunoblot analysis of BCL-X_L_ and RAS in H358, MIA PaCa-2 and SW837 xenograft tumors at the end of treatments as in **Fig. 2a-c** (*n* = 4 mice per group). **b,** Immunoblot analysis of BCL-X_L_, RAS, p-ERK, ERK, p-AKT, AKT, p-S6, S6, fCaspase-3, cCaspase-3, fPARP and cPARP in MIA PaCa-2 xenograft tumors after the mice were treated with sotorasib (Sot, 10 mg/kg/qd x 6 days, p.o.) and/or DT2216 (DT, 15 mg/kg/q4d x 2 doses, i.p.). The tumors were harvested 6 h and 24 h after the last dose of Sot and DT, respectively. **c,** Densitometric analysis of selected immunoblots normalized to equal loading control β-tubulin in **b. d,** Immunohistochemistry (IHC) of Ki67 and cleaved caspase-3 in H358 tumors from Fig. 2a. The images were captured at 200x magnification.

**Supplementary Table 1. Antibodies used in immunoblotting**

| **Antibody** | **Clone** | **Antibody isotype** | **Catalog #** | **Concentration** |
| --- | --- | --- | --- | --- |
| RAS | D2C1 | Rabbit IgG monoclonal | 8955S | 1:1000 |
| p-ERK T202/Y204 | _ | Rabbit IgG polyclonal | 9101S | 1:1000 |
| ERK | _ | Rabbit IgG polyclonal | 9102S | 1:1000 |
| p-AKT S473 | 193H12 | Rabbit IgG monoclonal | 4058S | 1:1000 |
| AKT | _ | Rabbit IgG polyclonal | 9272S | 1:1000 |
| p-S6 S240/244 | _ | Rabbit IgG polyclonal | 2215S | 1:1000 |
| S6 | 5G10 | Rabbit IgG monoclonal | 2217S | 1:1000 |
| p-EGFR Y1068 | D7A5 | Rabbit IgG monoclonal | 3777S | 1:1000 |
| EGFR | D38B1 | Rabbit IgG monoclonal | 4267S | 1:1000 |
| BCL-X_L_ | _ | Rabbit IgG polyclonal | 2762S | 1:1000 |
| BCL-2 | 50E3 | Rabbit IgG monoclonal | 2870S | 1:500 |
| BCL-W | 31H4 | Rabbit IgG monoclonal | 2724S | 1:500 |
| MCL-1 | D35A5 | Rabbit IgG monoclonal | 5453S | 1:1000 |
| BIM | C34C5 | Rabbit IgG monoclonal | 2933S | 1:1000 |
| p-BIM S69 | _ | Rabbit IgG polyclonal | 4581S | 1:1000 |
| BMF | E5U2J | Rabbit IgG monoclonal | 50542S | 1:1000 |
| PUMA | D30C10 | Rabbit IgG monoclonal | 12450S | 1:1000 |
| BID | _ | Rabbit IgG polyclonal | 2002S | 1:1000 |
| NOXA | D8L7U | Rabbit IgG monoclonal | 14766S | 1:1000 |
| BAX | _ | Rabbit IgG polyclonal | 2772S | 1:1000 |
| BAK | D4E4 | Rabbit IgG monoclonal | 12105S | 1:1000 |
| Full-length PARP | 46D11 | Rabbit IgG monoclonal | 9532S | 1:1000 |
| Cleaved PARP | D64E10 | Rabbit IgG monoclonal | 5625S | 1:1000 |
| full-length caspase-3 | _ | Rabbit IgG polyclonal | 9662S | 1:1000 |
| cleaved caspase-3 | _ | Rabbit IgG polyclonal | 9661S | 1:1000 |
| β-tubulin | _ | Rabbit IgG polyclonal | 2146S | 1:3000 |
| Secondary antibody |  | Anti-rabbit IgG, HRP | 7074S | 1:3000 |

**Footnotes:** All the antibodies were purhcased from Cell Signaling Technology, Danvers, MA.

**SUPPLEMENTARY References**

1. Khan S, Zhang X, Lv D, Zhang Q, He Y, Zhang P, et al. A selective BCL-X L PROTAC degrader achieves safe and potent antitumor activity. Nat Med. 2019;25(12):1938-47.
2. Thummuri D, Khan S, Underwood PW, Zheng P, Wiegand J, Zhang X, et al. Overcoming Gemcitabine Resistance in Pancreatic Cancer Using the BCL-X L Specific Degrader DT2216. Mol Cancer Ther. 2021. doi: 10.1158/1535-7163.MCT-21-0474. Online ahead of print.
